# Homogeneous *B*_1_^+^ for bilateral breast imaging at 7 T using a five dipole transmit array merged with a high density receive loop array

**DOI:** 10.1002/nbm.4039

**Published:** 2018-11-29

**Authors:** Erwin Krikken, Bart R. Steensma, Ingmar J. Voogt, Peter R. Luijten, Dennis W.J. Klomp, Alexander J.E. Raaijmakers, Jannie P. Wijnen

**Affiliations:** ^1^ Department of Radiology University Medical Center Utrecht Utrecht The Netherlands; ^2^ Department of Biomedical Engineering Eindhoven University of Technology Eindhoven The Netherlands

**Keywords:** 7 T, breast imaging, dipole antenna, high field

## Abstract

To explore the use of five meandering dipole antennas in a multi‐transmit setup, combined with a high density receive array for breast imaging at 7 T for improved penetration depth and more homogeneous *B*_1_ field. Five meandering dipole antennas and 30 receiver loops were positioned on two cups around the breasts. Finite difference time domain simulations were performed to evaluate RF safety limits of the transmit setup. Scattering parameters of the transmit setup and coupling between the antennas and the detuned loops were measured. In vivo parallel imaging performance was investigated for various acceleration factors. After RF shimming, a *B*_1_ map, a *T*_1_‐weighted image, and a *T*_2_‐weighted image were acquired to assess *B*_1_ efficiency, uniformity in contrast weighting, and imaging performance in clinical applications. The maximum achievable local SAR_10g_ value was 7.0 W/kg for 5 × 1 W accepted power. The dipoles were tuned and matched to a maximum reflection of −11.8 dB, and a maximum inter‐element coupling of −14.2 dB. The maximum coupling between the antennas and the receive loops was −18.2 dB and the mean noise correlation for the 30 receive loops 7.83 ± 8.69%. In vivo measurements showed an increased field of view, which reached to the axilla, and a high transmit efficiency. This coil enabled the acquisition of *T*_1_‐weighted images with a high spatial resolution of 0.7 mm^3^ isotropic and *T*_2_‐weighted spin echo images with uniformly weighted contrast.

**Abbreviations used**
AP: anterior–posterior
FH: feet‐head
FOV: field of view
RL: right–left
RMS: root mean square
SAR: specific absorption rate
SENSE: sensitivity encoding
TSE: turbo spin echo

## 1 INTRODUCTION

Imaging of the breast at 7 T is of interest due to the increased spectral and spatial resolution, enabling characterization of breast cancer and monitoring of treatment. However, breast imaging at 7 T is challenging mainly due to severe non‐uniformities in RF transmit (*B*_1_^+^) fields^1^, ^2^, ^3^ and limited penetration depth. A homogeneous *B*_1_^+^ field is particularly important for MR sequences such as turbo spin echo (TSE), which can still not compete with the clinical standard at 3 T.^4^ One approach to overcome *B*_1_ inhomogeneity is forced current excitation as demonstrated in previous studies.^5^, ^6^, ^7^ Another approach is the use of a multi‐transmit setup where RF shimming can be used to improve transmit uniformity for every subject.^1^, ^8^

A wide variety of breast MRI studies at 7 T have been presented with various RF coil setups.^7^, ^9^, ^10^, ^11^, ^12^, ^13^, ^14^, ^15^, ^16^, ^17^ Although imaging performance in the anterior part of the breast is generally good, clinical usability might be impeded by limited penetration depth towards the pectoral muscle, which is a frequently occurring challenge. To our knowledge, previous work on 7 T breast imaging has not shown bilateral *T*_2_‐weighted imaging or a field of view (FOV) reaching the axillary lymph nodes; two important measures that are essential for translation of the 3 T clinical exam to 7 T. Next to a uniform *B*_1_^+^, *B*_1_^+^ efficiency is important for dynamic contrast enhanced (DCE) MRI, where short but high flip angles are needed. Next to this, *T*_2_ relaxation time in breast tissue is short, so shorter 180° pulses are needed. Moreover, the bandwidth, for example, of pulses in MRS will be affected by the *B*_1_^+^ efficiency causing chemical shift displacements.

It has been demonstrated that dipole antennas^18^ have enhanced field uniformity and penetration depth compared with loop coils,^19^ which are often used in other breast coil designs. Since these are the main issues with breast imaging at 7 T, we explored whether the use of five meandering dipole antennas^20^, ^21^ (based on fractionated dipole antennas) in combination with 30 receive loops helps to improve the transmit efficiency, *B*_1_^+^ homogeneity and penetration depth. Specific absorption rate (SAR) and *B*_1_^+^ simulations are performed and compared with in vivo measurements to evaluate RF safety limits of this design.

## 2 SUBJECTS AND METHODS

### 2.1 Hardware

**Figure 1:**
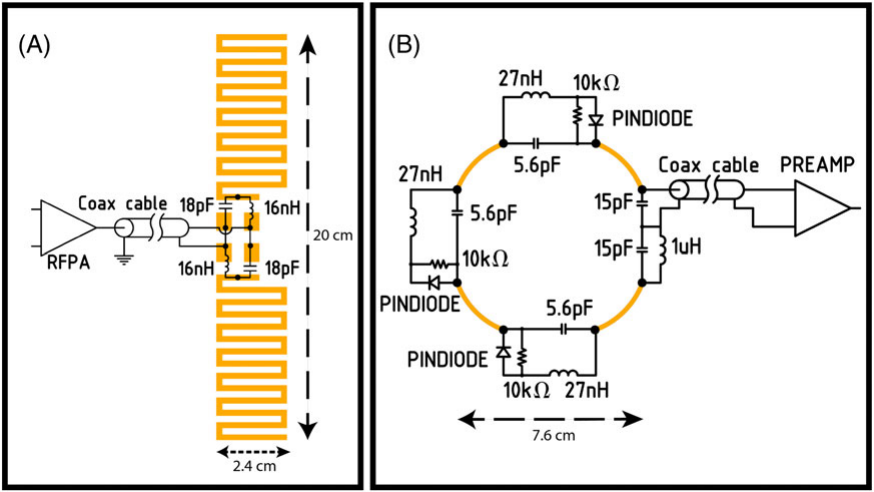
Electric circuitry of a meandering dipole antenna (A) and the electric circuitry of the receive loops (B). The black dots indicate the points where the lumped elements are soldered onto the printed circuit boards

**Figure 2:**
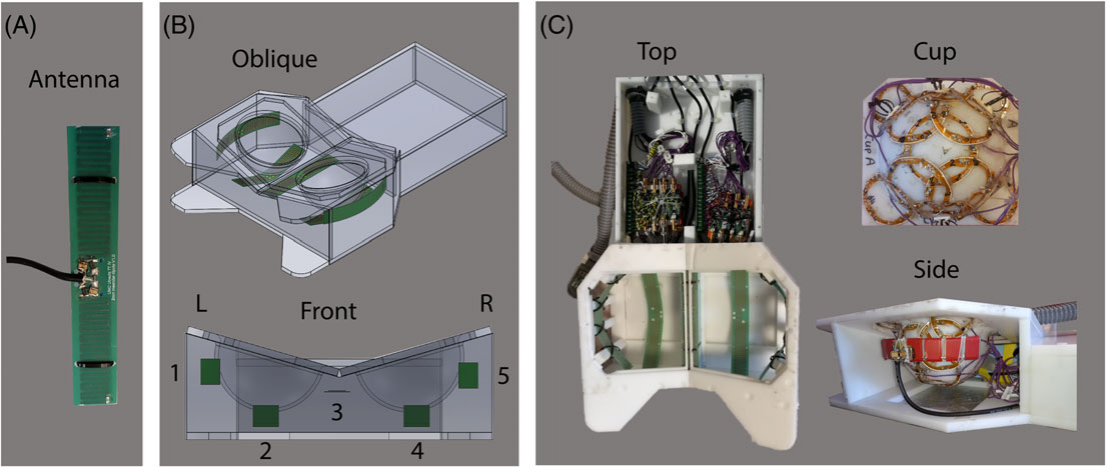
Setup of the breast coil. A, One of the five meandering dipole antennas used in the breast coil. B, Oblique and front views of the schematic design of the breast coil, where the placement of the dipole antennas is shown in green. C, Different views (top, cup and side view) of the hardware inside the coil

Five meandering dipole antennas were connected to five transmit channels of an eight channel multi‐transmit setup (8 × 2 kW peak power amplifiers, CPC, New York, USA) on a 7 T MR system (Philips, Cleveland, OH, USA). The design of the meandering dipole antenna was modified to tune the dipole antennas to resonance at a length of 20 cm, in order to fit next to the cups around the breasts and to match the geometry of the previous in‐house built coil^14^ so associated pillows and pads could be reused. Figure 1 shows the electrical circuit of the meandering dipole antennas (Figure 1A), which was identical for all antennas and the receive loops (Figure 1B). The dipole legs have a meandering geometry (Figure 1A), in order to maintain resonance in a loaded situation for the given length and to reduce SAR levels.^20^, ^21^, ^22^ The antenna and the positioning of the antennas are schematically shown in Figure 2A and 2B. The dipole antennas were manufactured on printed circuit boards from the material FR‐4 (Eurocircuits, Mechelen, Belgium). Ideally, the position of the antennas should be parallel to the receive loops to minimize coupling; however, due to the geometry of breast this was not possible for all coils. The chosen solution is a tradeoff between practical geometric position and minimized coupling.

Thirty receiver loops were tuned at 298 MHz and positioned on two cups around the breasts: 15 receivers with a diameter of 7.6 cm for each cup. To protect the volunteers from potential electric hazards, a 1 cm layer of electrically isolating material is placed between the receive circuitry and the volunteers. Each loop is individually shaped to match the geometry of the cups (Figure 2C). Decoupling of the loops was performed by overlapping of the adjacent loops and by preamplifier decoupling. Each receiver loop was actively detuned during the transmit phase using a parallel trap circuit with a PIN diode. The scattering parameters of the dipole antennas were measured with directional couplers during scanning.^23^ Coupling between the antennas in the presence of the detuned receiver loops as well as coupling between the antennas and 30 receive loops was measured on the bench. Proper functioning and isolation of the receive loops was determined from the noise correlation matrix.

### 2.2 Simulation

Finite difference time domain simulations (Sim4Life, Zurich Med Tech, Zurich, Switzerland) were performed to evaluate transmit efficiency and RF safety limits of the setup assuming full decoupling to the receiver coils (see Supplementary File CoilModel.smash). These simulations were also performed on three adjusted human models of Ella of the virtual family,^24^ where the breasts were replaced by breast models to better correspond to the breast shape in prone position.^14^, ^25^ The breast models were segmented from *T*_1_‐weighted scans performed at 7 T with and without fat suppression from three volunteers with different breast sizes (small/medium/large) to account for differences in coil loading. Scan parameters were FFE, *T*_R_ = 4.4 ms, *T*_E_ = 1.91 ms, flip angle = 8°, sensitivity encoding (SENSE^26^) 4 × 2 (right–left, RL, × feet‐head, FH), FOV = 160 × 350 × 160 mm^3^ and voxel size = 0.7 × 0.7 × 0.7 mm^3^. For the fat‐suppressed image the same parameters were used, including a 1–3–3‐1 binomial spectral spatial RF pulse for fat suppression. Glandular tissue and fat tissue were segmented using iSeg (Medical Image Segmentation Tool Set, Zurich Med Tech) and the corresponding dielectric permittivity and conductivity were assigned. The worst‐case SAR was calculated as the maximum sum of the modulus of all quality matrix (*Q*‐matrix) entries,^27^ yielding the worst‐case SAR for an equal input power of 1 W on every channel. This value was used to derive average power limits per transmit channel for imaging applications, based on a 10 g averaged SAR limit of 20 W/kg in the trunk for the first level controlled mode.^28^ Safety simulations performed for this coil are validated by means of comparing measured and simulated *B*_1_^+^ maps. Worst‐case SAR values are used to determine safe power limits resulting in conservative SAR estimates.

### 2.3 Measurements

Safe average power limits were derived from simulations. Several safety measures are implemented on the scanner system in order to assure a duty cycle in which the defined average power is never exceeded. A safety check is executed before every scan, checking the *T*_R_ and the necessary peak power and comparing the resulting average power against the power limit that is set in the software. Additionally, continuous power measurements are conducted during the scan with the directional couplers to ensure that average power limits are not exceeded. RF pulse width and duty cycle differ per sequence, but are forced to never exceed the average power limit.

After informed consent, two healthy female volunteers were scanned. For both volunteers, the phases and amplitudes of the individual transmit channels were optimized to obtain a uniform signal in the breast (RF shimming).^1^, ^29^ For this purpose, spoiled gradient echo images were acquired at a low flip angle for every single transmit channel. Magnitude and phase results were exported to a post‐processing tool (MATLAB, MathWorks, Natick, MA, USA). This tool is used to combine the single transmit channel images into a “shimmed” combined image using different amplitudes and phases for every transmit channel. A region of interest (ROI) was drawn in the breast, after which a constrained minimization was used to optimize the channel weights. The goal of this procedure was to minimize the coefficient of variation in the ROI, while maintaining a minimum threshold of 40% and a maximum threshold of 100% for the drive amplitude. To assess the *B*_1_^+^ performance of the coil, a *B*_1_^+^ map was acquired using the dual refocusing echo acquisition mode method (DREAM^30^) with a nominal *B*_1_ of 12 μT. For the *B*_1_^+^ map acquisition, RF shimming resulted in a peak input power of 710 W per channel except for Channel 3 (middle antenna), which was driven at 126 W. Additional scan parameters were *T*_R_ = 7.0 ms, *T*_E_ = 1.97 ms and 2.4 ms, flip angle = 10° and 60°, slice spacing = 21 mm, and FOV = 320 × 520 × 60 mm^3^ with a resolution of 3.0 × 5.0 × 6.0 mm^3^. The acquired *B*_1_ map was compared with a simulated *B*_1_ map with identical excitation phase and amplitude per channel to validate the EM simulations. The mean *B*_1_^+^ and coefficient of variation (=standard deviation/mean) has been calculated in two ROIs: the first (ROI1) includes both breasts to the pectoralis muscle boundary, and the second (ROI2) has been set for the entire FOV measured, including the pectoral muscle and reaching into the axilla.

Parallel imaging performance was investigated for the SENSE algorithm^26^ and represented by geometry factor (g‐factor) maps. ReconFrame (Gyrotools, Zürich, Switzerland) was used to calculate g‐factor maps for different acceleration factors from *R* = 1 to *R* = 4 in the RL direction and *R* = 1 to *R* = 3 in the anterior–posterior (AP) direction by reconstructing the image undersampling in *k*‐space. SNR maps were calculated in SNR units.^31^, ^32^

To demonstrate clinical feasibility, a 3D *T*_1_‐weighted image was acquired (FFE, *T*_R_ = 7.14 ms, *T*_E_ = 3.23 ms, flip angle = 8°, nominal *B*_1_ = 9 μT, SENSE 4 × 2 (RL × FH), FOV = 160 × 351 × 160 mm^3^, resolution = 0.7 × 0.7 × 0.7 mm^3^) using a water selective 1–4–6‐4‐1 spectral spatial RF pulse for fat suppression in 111 s. Also, a single slice *T*_2_‐weighted TSE image (*T*_R_ = 10 000 ms; *T*_E_ = 90 ms; echo‐spacing = 10 ms; flip angle = 90°; refocusing flip angle = 180°; nominal *B*_1_ = 12 μT; FOV = 250 × 421 mm^2^; resolution = 0.7 × 0.7 × 3 mm^3^; TSE factor = 17) was acquired in 2 min 30 s.

## 3 RESULTS

### 3.1 Hardware

**Figure 3:**
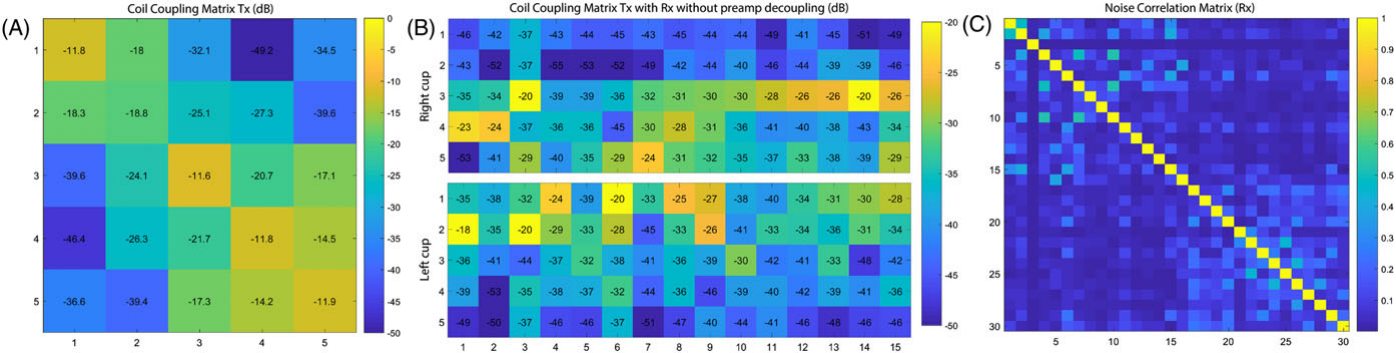
A, Inter‐element coupling of the transmit dipole array with a maximum inter‐element coupling of −14.2 dB. B, Coil coupling matrix of the five dipole antennas with the receive loops of both cups with a maximum coupling of −18.2 dB. C, Noise correlation matrix of the 30 receive loops with a mean of 7.83 ± 8.69% and a maximum correlation of 52.9%

Inter‐element coupling between the dipole antennas is generally low. The maximum coupling of the transmit dipole array was −14.2 dB between Elements 4 and 5, while maximum reflection was −11.6 dB in Element 3 (Figure 3A) for a representative subject. The maximum coupling of the transmit dipole with the receive loops without preamp decoupling occurred between Element 2 and Receive Loop 1 from the left cup and was −18.2 dB (Figure 3B). The noise correlation matrix of the 30 receive loops has a mean of 7.8 ± 8.7% with a maximum correlation of 52.9% (Figure 3C).

### 3.2 Simulation

**Figure 4:**
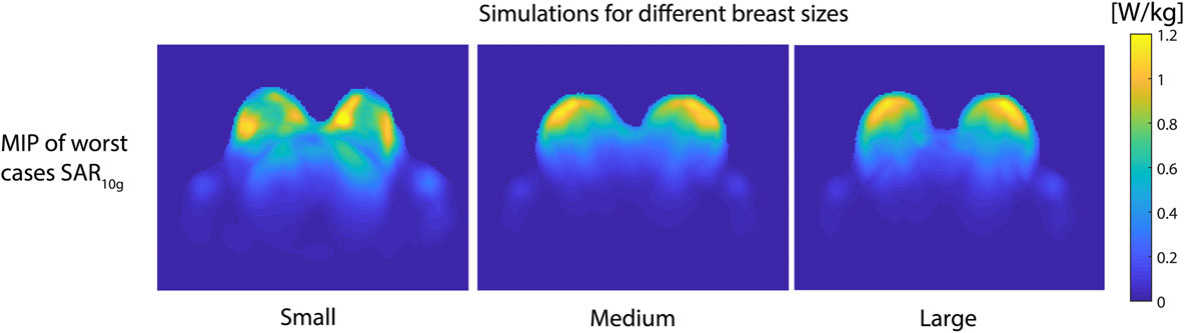
Simulation of maximum possible SAR distribution on Ella (Virtual Family) of the transmit elements with adapted breast models with different sizes (small/medium/large). The breast models were segmented from *T*_1_‐weighted scans performed at 7 T with and without fat suppression with a previously built bilateral breast coil. The color bar indicates the local 10 g averaged SAR (W/kg)

The maximum achievable local SAR value is 7.0 W/kg for 5 × 1 W accepted power (Figure 4) in the adjusted Ella model of the Virtual Family in prone position. Considering the local SAR limit of 20 W/kg in the trunk in first level controlled mode, an average power of 2.9 W/channel can be used at maximum.

### 3.3 Measurements

**Figure 5:**
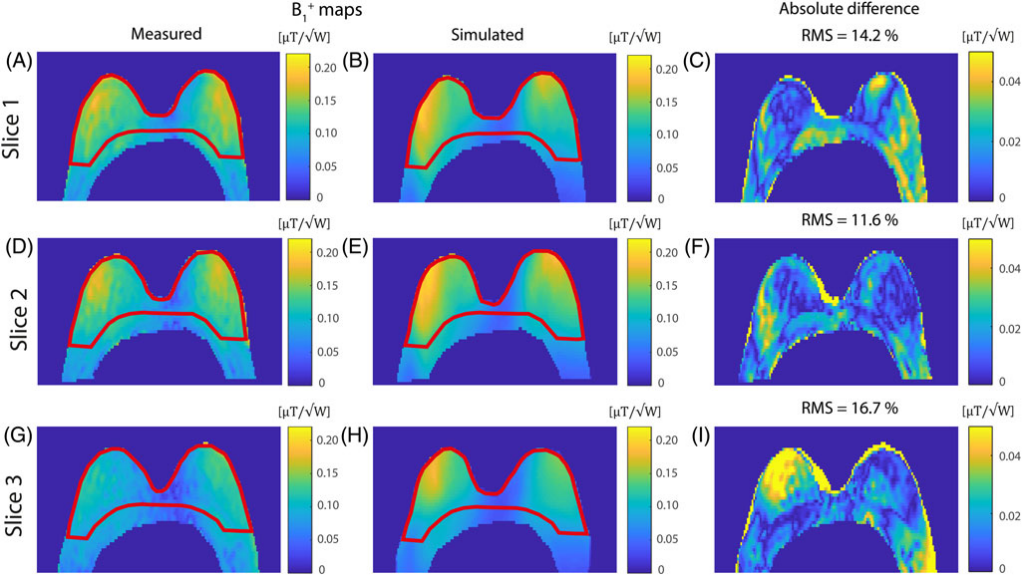
Three transversal slices of the measured *B*_1_^+^ map (same volunteer as ‘small’ in Figure 4) compared with the simulated *B*_1_^+^ map with the same shim settings, resulting in the absolute difference map with a mean RMS of 14.2%. The measured *B*_1_^+^ map was acquired with the DREAM method (*T*_R_ = 7.0 ms; *T*_E_ = 1.97 ms and 2.4 ms; flip angle = 10° and 60°, FOV = 320 × 520 × 60 mm^3^ with a resolution of 3.0 × 5.0 × 6.0 mm^3^ and a nominal *B*_1_ of 12 μT). The mean *B*_1_^+^ has been measured in two ROIs in the measured *B*_1_ map, where ROI1 includes both breasts to the pectoralis muscle boundary (shown in red) and ROI2 has been set for the entire FOV shown (including the pectoral muscle and reaching into the axilla). Within ROI1 a mean *B*_1_^+^ of 0.12 μT/√W, coefficient of variation 20%, was measured, and in ROI2 a mean *B*_1_^+^ of 0.11 μT/√W, coefficient of variation 27%

The acquired DREAM *B*_1_^+^ map (Figure 5) shows that, with successful RF shimming, the transmit fields of the antennas penetrate through the breasts completely. The magnitude of the transmit field also remains relatively homogeneous throughout the breast: within ROI1 a mean *B*_1_^+^ of 0.12 μT/√W, coefficient of variation 20%, and in ROI2 a mean *B*_1_^+^ of 0.11 μT/√W, coefficient of variation 27% (Figure 5). Good correspondence between the measured *B*_1_^+^ and the simulated *B*_1_^+^ was found, with a mean root mean square (RMS) of 14.2% (Figure 5).

**Figure 6:**
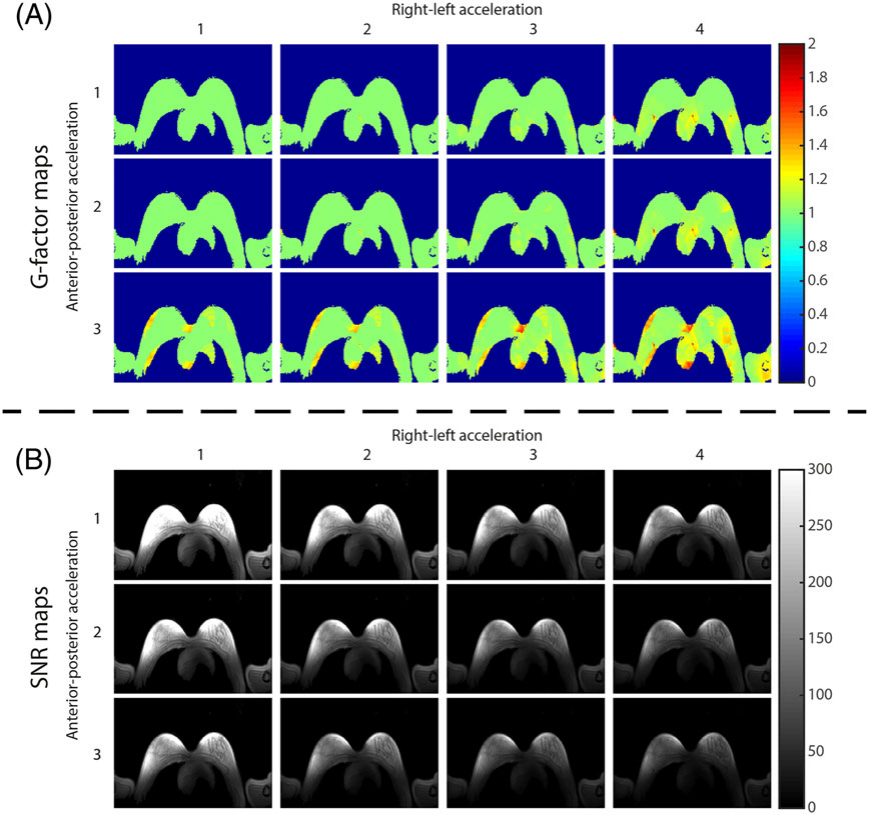
A transversal slice of g‐factor maps with different acceleration factors in RL direction and AP direction for the receive chain (A) and the corresponding SNR maps in SNR units (B)

The g‐factor maps for different acceleration factors for the receive loops are displayed in Figure 6A. High g‐factors (g‐factor ≥ 2.0) start to appear for acceleration factors higher than *R* = 4 in the RL direction and *R* = 3 in the AP direction. However, the average g‐factor is only 1.11 when moving to an acceleration factor of *R* = 4 × 3, with a maximum g‐factor of 1.87 near the heart. SNR decreases with increasing acceleration factor (Figure 6B).

**Figure 7:**
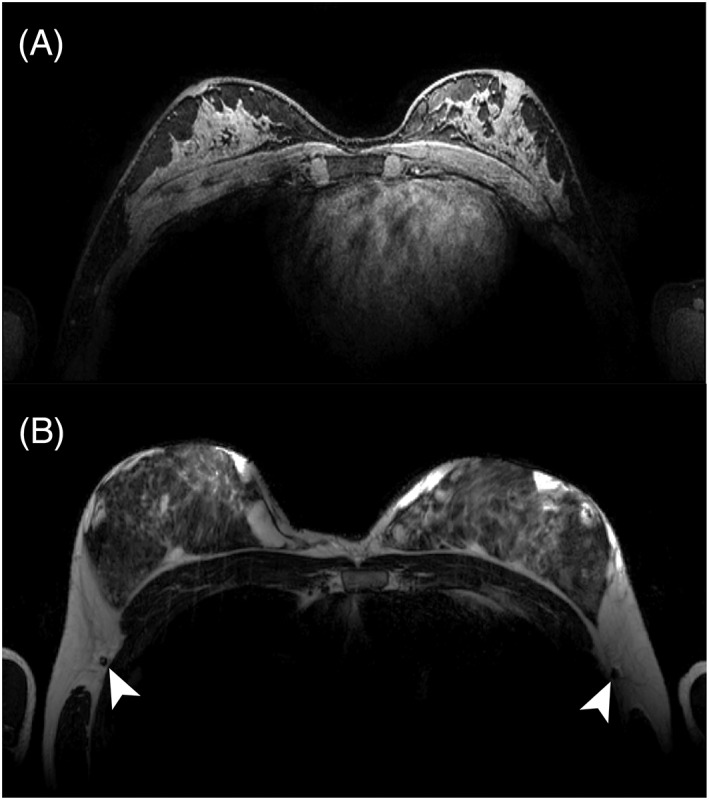
Examples of clinical scans acquired with the coil. A, A transversal slice of a fat‐suppressed *T*_1_‐weighted image (*T*_R_ = 7.1 ms; *T*_E_ = 3.2 ms; flip angle = 8°; resolution 0.7 mm^3^; SENSE 4 × 2 (RL × FH), 1–4–6‐4‐1 spectral spatial RF pulse for fat suppression). A total acceleration of *R* = 8 was used, compromising the SNR achieved in the axilla, yet high detail can be observed in the glandular tissue. B, A transverse *T*_2_‐weighted TSE image (*T*_R_ = 10 000 ms; *T*_E_ = 90 ms; flip angle = 90°; resolution = 0.7 × 0.7 × 3 mm^3^; TSE factor = 17) . Note the lymph nodes in the axilla depicted as hypo‐intense spots indicated by the white arrows

We were able to acquire ultra‐high resolution 3D fat‐suppressed *T*_1w_ images in 111 s (Figure 7A). Good fat suppression is achieved throughout the whole breast. A very high acceleration factor of *R* = 8 was used, compromising the SNR achieved in the axilla, yet high detail can be observed in the glandular tissue. Moreover, a *T*_2_‐weighted TSE image (Figure 7B) was acquired with clear contrast between fat tissue and glandular tissue. Note that the lymph nodes in the axilla can be observed as hypo‐intense spots.

## 4 DISCUSSION

We have demonstrated a new breast coil design using a multi‐transmit system and five meandering dipole antennas around the breasts in combination with 30 receive loops. With this setup we were able to achieve a relatively homogeneous *B*_1_^+^ throughout the breast. However, the assessment of homogeneity in the superior–inferior direction can be improved, as only three slices were obtained around the center of the breasts. Previously presented coils^7^, ^9^, ^10^, ^11^, ^12^, ^13^, ^14^ show that penetration depth is often limited to the pectoral muscle. However, it is important for clinical breast imaging that lymph nodes inside the axilla are visible on the images as well. With the use of meandering dipole antennas in this setup, we were able to acquire images with a large FOV due to the high penetration depth of the antennas. The achieved *B*_1_^+^ and homogeneity allowed us to acquire bilateral *T*_2_‐weighted images of the breasts, which has been impossible with other bilateral designs at 7 T so far, yet crucial for clinical practice. Only a single slice for the *T*_2_‐weighted image could be acquired within the SAR limits in this study. Future work will include an optimization of the *T*_2_‐weighted sequence in terms of *T*_R_ and RF pulses in order to acquire more slices in the same time, which has already been shown in the prostate.^33^

Simulation of the breast coil ensured patient safety; however, worst‐case SAR values were used to determine safe power limits, resulting in a conservative SAR estimate. Future work will focus on the application of more realistic SAR levels, which will require a study of the inter‐subject variability and further validation efforts using deep learning.^34^

In this study, the worst‐case SAR assessment assumes equal and unitary input power on every transmit channel. In our current safety procedure, power limits are defined on a per channel basis. In the case where amplitude shimming is applied, the drive amplitude of a single channel will be scaled down but the power limit will stay the same. This ensures conservative setting for the power limits, in which SAR can never exceed the SAR as calculated in our models.

After impedance matching of the antenna array, a worst‐case inter‐element coupling of −14.2 dB and a worst‐case reflection coefficient of −11.7 dB are demonstrated. Although it is possible to improve the worst‐case reflection coefficient for a single subject, a tradeoff between robustness to load and an excellent reflection coefficient had to be chosen, leading to the current performance. The measured noise matrix showed good isolations between the receiver array channels, and the measured g‐factor maps showed the feasibility of parallel imaging with the developed RF array system. High acceleration factors, up to an acceleration of *R* = 4 × 3 (RL × AP directions) were achieved while preserving image quality, as no unfolding artifacts within the breast were observed (Figure 5). The g‐factor maps do show higher g‐factors appearing in the axilla and in the arms of the volunteer. This is due to the fact that the receive loops are located around the breasts and do not receive signal originating from beyond the breast, while the meandering dipole antennas have a much larger penetration depth. The optimal way to avoid signal loss is to combine the signals from loops and antennas in reception, resulting in a total of 35 receive elements. Unfortunately, the MR system architecture currently does not allow more than 32 element receive.

The currently proposed transmit setup places five dipole antennas on the anterior side of the subject. Commonly used 7 T body transmit setups use eight elements placed concentrically around the subject. It would be possible to place additional transmit elements on the posterior side of the subject. However, it is demonstrated in this work that the current setup has sufficient penetration depth to cover the entire breasts. Adding more antennas on the posterior side of the subject could result in SAR peaks in the back muscles, and would add to the overall complexity of the setup, so this was not considered for this coil array.

The decoupling value between the transmit antennas and the receive loops of −18 dB is measured without the presence of pre‐amplifier decoupling. Therefore, the actual decoupling is estimated to be below −25 dB, which is sufficient to protect the preamplifiers during transmit. All scattering parameters remain well below −10 dB, indicating that more than 90% of the emitted power is accepted and therefore 95% of the potential *B*_1_^+^ is reached. Additional reduction in reflection may be obtained, but would provide at maximum only a few percent additional *B*_1_^+^ and was therefore left for a future improvement cycle.

An often occurring challenge with breast imaging at 7 T is dealing with the inhomogeneity of *B*_1_^+^, decreasing from the nipple towards the pectoral muscle. In our bilateral coil setup, the position and the penetration depth of the antennas creates a homogenous *B*_1_^+^ in the AP direction throughout the breasts (coefficient of variation 20% within the breasts; ROI1 in Figure 5). Though hard to compare with other work, Kim et al.^10^ reported a coefficient of variation of 22% for one transversal slice and 31% in the entire volume, and Brown et al.^15^ reported a coefficient of variation of 24% in one transversal slice. The observed inhomogeneity in the RL direction is probably due to interference of the *B*_1_^+^ fields of the different antenna elements. We have observed this asymmetry in all the volunteers and therefore we think this is independent of the breast size. To achieve a more homogeneous *B*_1_^+^ throughout both breasts, more advanced calibration of the transmit fields is necessary, yet RF shimming only cannot solve this problem. Adding more antennas to the coil array may help to increase uniformity in the RL direction. Next to this, tailored RF pulses may be able to compensate for the *B*_1_^+^ inhomogeneity: for example, tilt optimized flip uniformity pulses^35^ or tailored excitation in 3D with spiral nonselective (SPINS) RF pulses.^36^ A next step in breast imaging at 7 T would be to incorporate tailored RF pulse designs into the clinical protocol.

## 5 CONCLUSION

We constructed a new breast coil with five meandering dipole antennas and 30 receive loops for 7 T. The coil can generate sufficient *B*_1_^+^ over a larger FOV in the breasts, which makes it possible to acquire *T*_2_‐weighted images and to image the axillary lymph nodes. This is an important step towards translating routinely used breast imaging protocols from 3 T to 7 T, whilst benefitting from the increased spectral and spatial resolution at 7 T.

## Supporting information

Supporting info item
